# Whether G-CSF administration has beneficial effect on the outcome after assisted reproductive technology? A systematic review and meta-analysis

**DOI:** 10.1186/s12958-016-0197-2

**Published:** 2016-09-22

**Authors:** J. Zhao, B. Xu, S. Xie, Q. Zhang, Y. P. Li

**Affiliations:** Department of Reproductive Medicine, Xiangya Hospital, Central South University, 87 Xiangya Road, Changsha City, Hunan Province 410008 People’s Republic of China

**Keywords:** Granulocyte colony-stimulating factor (G-CSF), Implantation rate, Pregnancy rate, Assisted reproductive technology (ART)

## Abstract

**Background:**

Previous studies have explored the effect of granulocyte colony stimulating factor (G-CSF) administration on the outcome of assisted reproductive technology (ART), and came into controversial conclusions. The present meta-analysis aims to assess whether G-CSF administration has beneficial effect on the outcome after ART.

**Method:**

The electronic databases Pubmed, Embase and Google Scholar were searched up to May 2016. Articles that studied the effect of G-CSF administration on the outcome after ART were included in the present meta-analysis. Odds ratio (OR) with 95 % confidence interval (95 % CI) were calculated to assess the effect of G-CSF administration on the outcome after ART. The outcomes of interest were implantation rate (IR) and pregnancy rate (PR).

**Results:**

Four cohort studies with 1101 embryos transplantation assessed the effect of G-CSF administration on IR and 6 studies with 621 cycles assessed the role of G-CSF administration in PR. Meta-analysis did not found an increased embryo IR in G-CSF administration cycles [OR 1.59 (95 % CI 0.74–3.41). whereas the PR with G-CSF administration was significantly higher compared with cases without G-CSF administration [OR 2.03 (95 % CI 1.19–3.46)]. Additionally, we found that G-CSF administrated subcutaneously resulted in significantly higher PR [OR 3.12 (95 % CI 1.67–5.81)] and IR [OR 2.82 (95 % CI 1.29–6.15)] compared with control group, whereas G-CSF administrated via local uterine infusion had no beneficial effect on the PR [OR 1.42 (95 % CI 0.91–2.24)] and IR [OR 1.10 (95 % CI 0.76–1.60)] after ART.

**Conclusions:**

G-CSF administration may have beneficial effect on clinical pregnancy outcome after ART. Subcutaneous injection may be an optimal route of G-CSF administration. Further cohort studies are required to explore the mechanisms undergone the effect and investigate the best route and dose of G-CSF administration.

**Electronic supplementary material:**

The online version of this article (doi:10.1186/s12958-016-0197-2) contains supplementary material, which is available to authorized users.

## Background

Successful embryo implantation required good-quality embryos and receptive endometrium. Studies indicated that endometrial receptivity should responsible for 2/3 embryo implantation failure. As shown in our previous papers, endometrial thickness is an important index evaluating endometrial receptivity [1, 2]. And many studies believed that endometrial thickness below a threshold is associated with embryo implantation failure and reduced pregnancy rate [3–8].

So far, many therapies have been attempt to enhance the endometrial thickness and improve the endometrial receptivity, such as extending estrogen administration [9], low-dose aspirin [10], combination pentoxifylline and tocopherol [11], vaginal sildenafil citrate [12], and stem cells treatment [13, 14]. These treatments have improved endometrial receptivity and increased implantation and pregnancy rate in ART cycles in some extent. However, many cases still remain unresponsive.

Lately, granulocyte colony-stimulating factor (G-CSF) has been used in the treatment of thin endometrium. Five years ago, Gleicher et al applied G-CSF in 4 infertile women with unresponsive thin endometrium for the first time, and resulted in successful pregnancy [15]. Subsequently, several studies have explored the effect of G-CSF administration on the outcome of ART with thin endometrium [16–20], or with repeated IVF failure (RIF) [21, 22]. And one study implied G-CSF in unselected women who received in vitro fertilization (IVF) treatment [23]. These studies came into controversial conclusions, so both clinicians and infertile women are in an awkward position of whether the G-CSF should be given.

In the present review, we aim to further evaluate whether G-CSF administration has beneficial effect on the outcome of ART with thin endometrium or with RIF and perform a systematic review and meta-analysis of the available literatures.

## Methods

### Identification of the literature

The electronic databases Pubmed, Embase and Google Scholar were searched up to May 2016. We included papers which explore the effect of G-CSF administration on thin endometrium and/or clinical outcome after ART treatment. The keywords were as follows: [(“G-CSF” or “CSF” or “granulocyte-stimulating factor”) and (“thin endometrium” or “endometrium receptivity” or “endometrial receptivity” or “endometrium thickness”) and (“in vitro fertilization” or “IVF” or “intracytoplasmic sperm injection” or “ICSI” or “frozen embryo transfer” or “FET” or “infertility treatment” or “assisted reproductive technology”)]. There were no limitations on the time and the type of the publications.

Titles and abstracts of all identified studies were screened and the full paper of the preselected articles was reviewed by two researchers. A 2 × 2 table was extracted from the articles. Any discrepancies between the two reviewers were resolved by group discussion.

### Eligibility criteria

Cohort studies with control group and RCTs investigating the effect of G-CSF administration on the endometrium and/or ART outcomes were considered to be eligible for inclusion. Studies without control group were excluded even if the content was related. Papers written in non-English were excluded.

The patient population comprised infertile women with all ages, receiving the IVF or intracytoplasmic sperm injection (ICSI) or frozen embryo transfer (FET) treatment. Cycles with donor oocyte/sperm and intrauterine abnormalities were excluded from analysis.

The main study outcomes were embryo implantation rate (determined by the number of gestational sacs at least 28 days after embryo transplantation based on the total number of embryos transferred per group), clinical pregnancy rate (gestational sac and fetal heart on ultrasound examination). All clinical outcomes were calculated per cycle.

### Quality assessment

Each selected study was scored for their relevance and methodological quality by using the Strengthening the Reporting of Observational Studies in Epidemiology (STROBE) checklists for Observational Studies. In addition, some information, such as sample size, study design, blinding, selection bias, information bias, attrition bias, and the stimulation protocol used were taken into consideration. Two reviewers completed the quality assessment, and any disagreements about inclusion were resolved by group discussion.

## Statistical analysis

A standard meta-analytic method was utilized to compare the studies which were included in this study and Odds Ratio (OR) with its 95 % CI was applied to express the combined result. Forest plots evaluated the heterogeneity of the studies graphically and l^2^ statistic quantified the heterogeneity between studies statistically. The heterogeneity was considered as low when the value was less than 50 % [24]. A random effect model or a fixed effect model was implied to evaluate a pooled OR and its 95 % CI. RevMan 5.0 was used to perform the statistical analysis (Cochrane Collaboration, Oxford, UK). The results were regard to be statistically significant when the *P* value was <0.05.

## Results

### Studies selection and characteristics

**Fig. 1:**
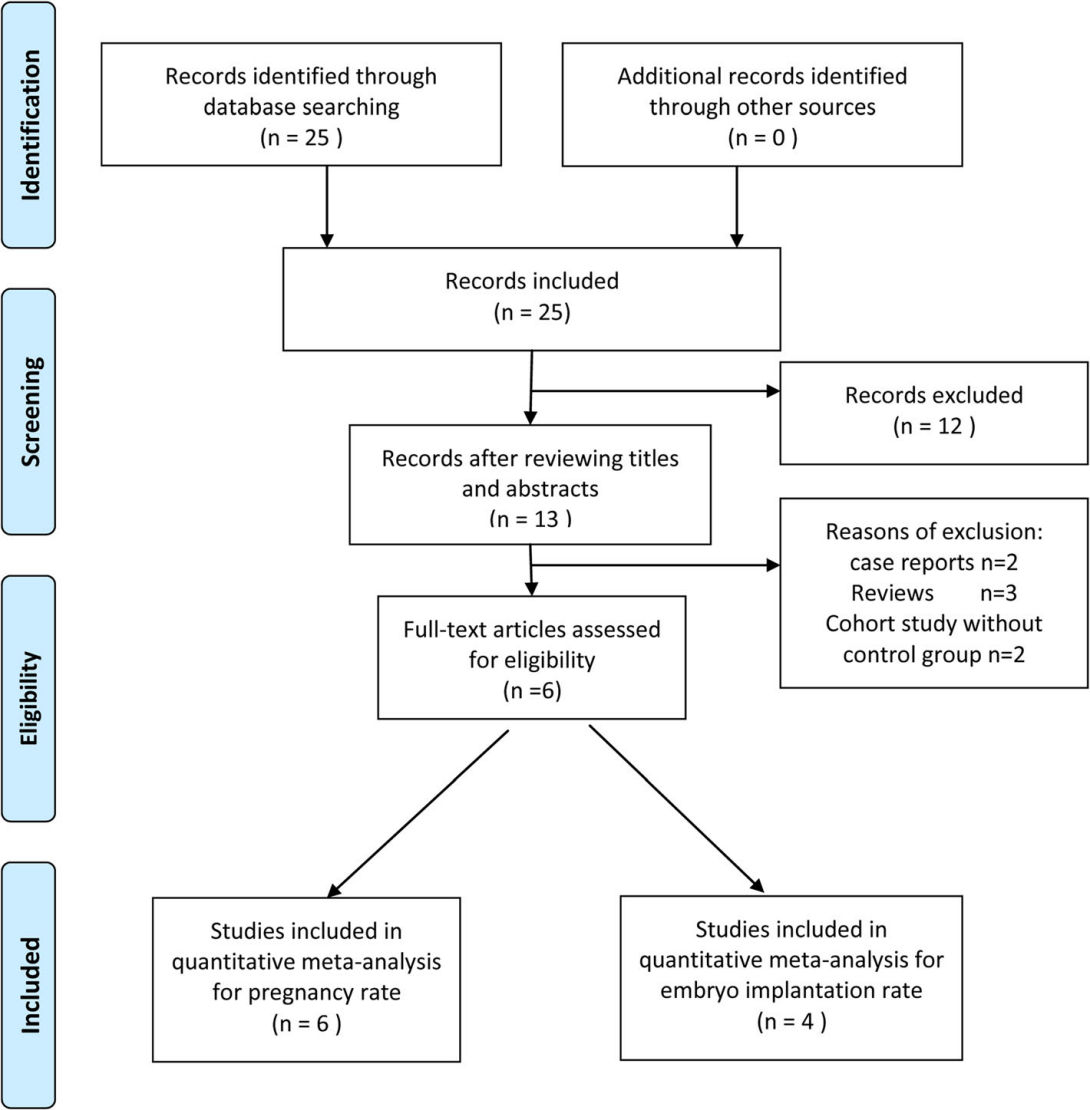
Flow chart showing study selection process

The search strategy yielded 25 citations. 12 citations were irrelevant and were excluded after reviewing the titles and the abstracts. Of the 13 remaining publications, seven were excluded and the reasons were as follows: two studies were case reports, three studies were reviews, and two studies were cohort studies without control groups (Fig. 1).

At last, six eligible studies were included in the review (two IVF studies, three FET studies, one IVF/FET study). All included studies comprising 621 cycles showed the effect of G-CSF administration on the pregnancy rate after ART with 172 pregnancies. Of these, 4 publications involved 1101 embryos transplantation also reported implantation rate.

Table 1 listed the characteristics of the included studies. Of these six studies, four studies were designed prospectively, one was retrospective and one was retrospective / prospective. Three of the papers evaluated the effect of G-CSF administration on outcome of ART cycles with thin endometrium, two of these studies assessed the influence of G-CSF administration on RIF, and one study investigated the efficacy of G-CSF usage in routine, unselected IVF cycles. G-CSF was administered subcutaneously in two studies and by transcervical intrauterine infusion in four studies.

**Table 1:** Characteristics of studies included in a systematic review and meta-analysis of G-CSF and pregnancy outcome after ART

Study		Type of study	Women	Treatment	Protocol	Dose and Rout of G-CSF administration	Outcome
2012	Scarpellini F	RCT	RIF	IVF	Not mentioned	300ug subcutaneously	P
2013	Yu Li	Retro	Thin endometrium	FET	Natural cycle; extended estrogen cycle; induced cycle	100ug uterine infusion	I, P, M
2014	Eftekhar M	Pro	Thin endometrium	FET	extended estrogen cycle	300ug uterine infusion	Chemical P, P
2014	Barad DH	RCT	Unselected	IVF/FET	Not mentioned	300ug uterine infusion	I, P
2015	Bin Xu	Retro/Pro	Thin endometrium	FET	Natural cycle/EMS	300ug uterine infusion	I, P, M, B
2016	Aleyasin A	RCT	RIF	IVF	GnRH-a long protocol	300ug subcutaneously	I, CP, P, EP

### Meta-analysis

**Fig. 2:**
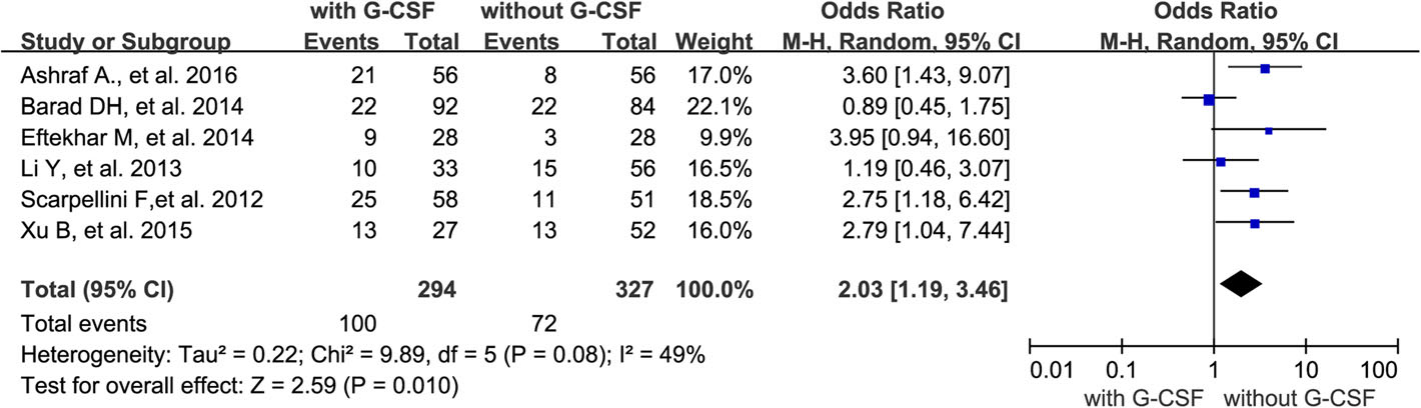
Forest plot showing the results of meta-analysis of studies evaluating the effect of G-CSF administration on pregnancy rate after ART

Six studies were included in the present review and meta-analysis to evaluate the G-CSF’s effect on PR after ART. We found a significant increased PR in infertile women who received the G-CSF administration compared with those without G-CSF. With a *P* value <0.1, the heterogeneity showed to be moderate without significance (l^2^ = 49 %, *P* = 0.08). The pooled OR with a random effects model was 2.03 (95 % CI 1.19–3.46, *P* = 0.010) (Fig. 2).

**Fig. 3:**
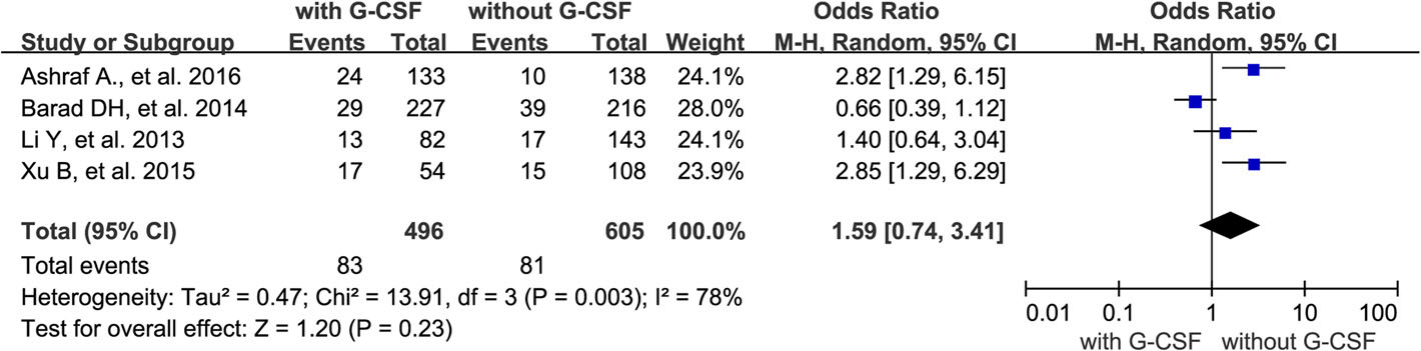
Forest plot showing the results of meta-analysis of studies evaluating the effect of G-CSF administration on embryo implantation rate after ART

Of these studies, 4 also analyzed the effect of G-CSF administration on implantation rate. The result of the meta-analysis indicated that there was similar implantation rate between G-CSF group and control group. The Q statistic *P*-value was below 0.05, indicating heterogeneity of the studies (l^2^ = 78 %, *P* = 0.003). The random effects model was implied and the combined OR was 1.59 (95 % CI 0.74–3.41, *P* = 0.23) (Fig. 3).

**Fig. 4:**
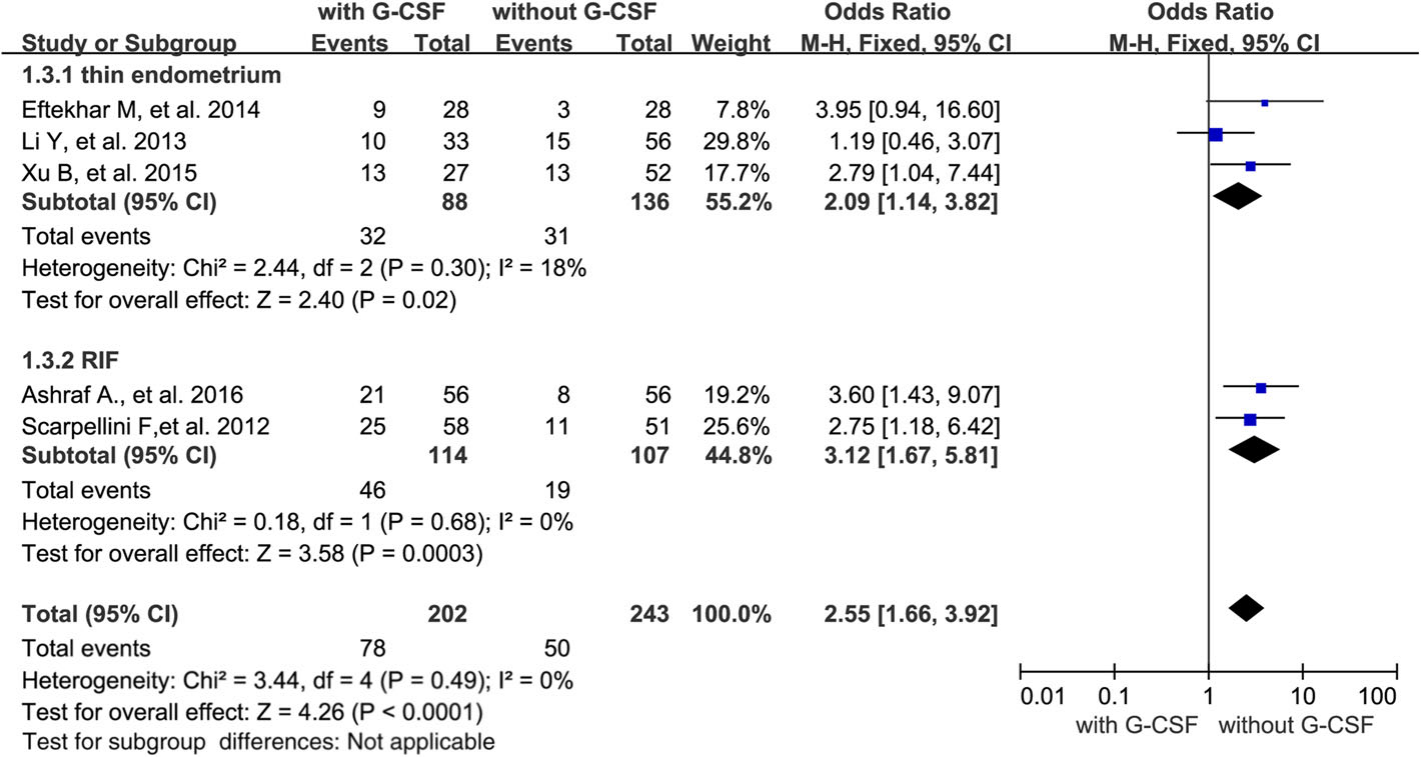
Forest plots showing the results of meta-analysis of studies evaluating the effect of G-CSF administration on pregnancy rate after ART cycles with thin endometrium or with RIF

In addition, we presented stratified results by the reason of G-CSF administration (thin endometrium *n* = 3; RIF *n* = 2; unselected women *n* = 1). As only one paper was related to unselected infertile women, we analyzed cycles with “thin endometrium” and “RIF”, separately. When evaluating the effect of G-CSF administration on PR, 3 studies were associated with thin endometrium and 2 studies were with RIF. The results indicated that a significant increased PR with G-CSF administration in both thin endometrium cycles (OR 2.09; 95 % CI 1.14–3.82, *P* = 0.02) and RIF cycles (OR 3.12; 95 % CI 1.67–5.81, *P* = 0.0003) (Fig. 4).

**Fig. 5:**
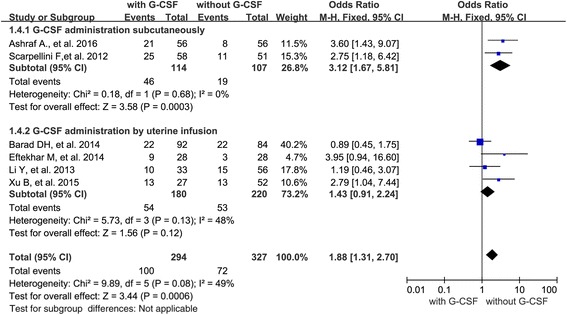
Forest plots showing the results of meta-analysis of studies evaluating the effect of G-CSF via different administration routes on pregnancy rate after ART cycles

When evaluating the IR in cycles with thin endometrium or RIF, 2 studies were with thin endometrium and only one study was with RIF. The result showed significant increased implantation rate when G-CSF was administrated in both thin endometrium cycles (OR 1.97; 95 % CI 1.14–3.42, *P* = 0.02) and the only one study which assessed the IR in RIF cycles showed beneficial effect of G-CSF administration on the embryo implantation (Fig. 5).

**Fig. 6:**
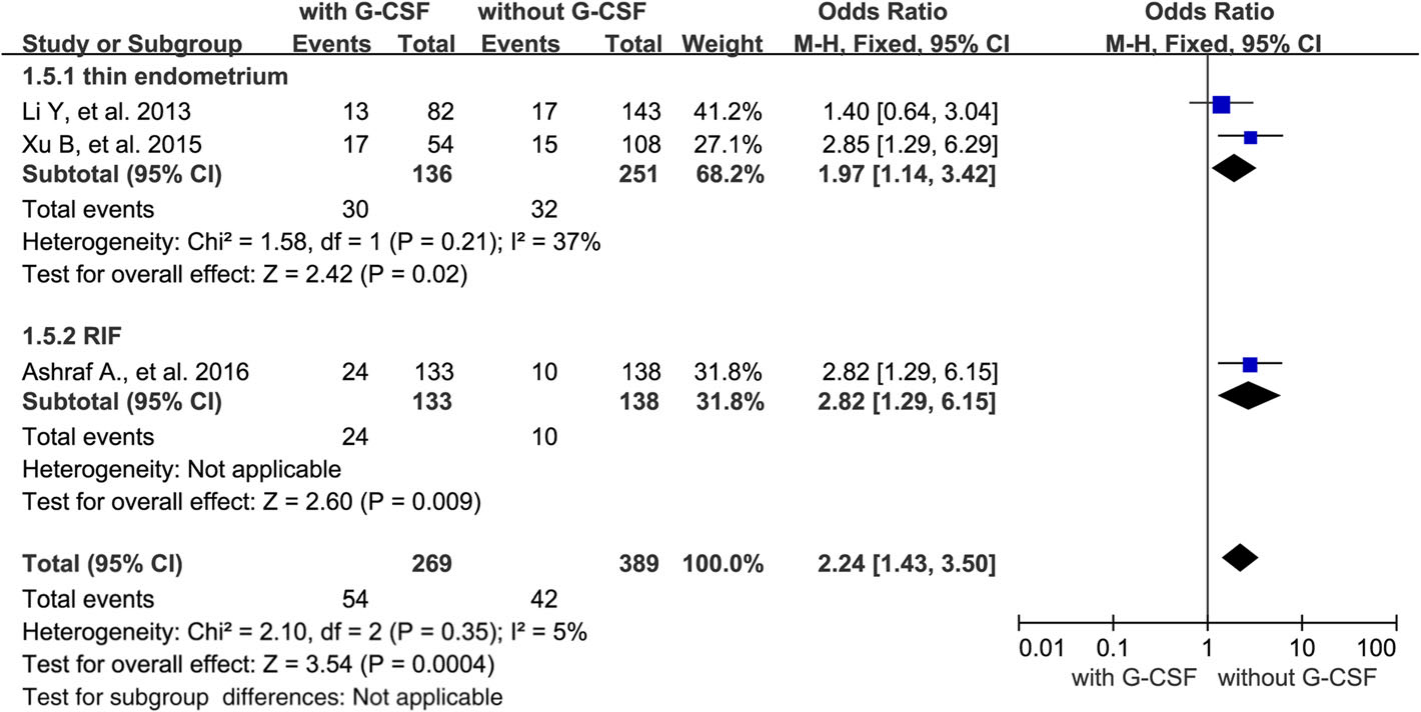
Forest plots showing the results of meta-analysis of studies evaluating the effect of G-CSF administration on embryo implantation rate after ART cycles with thin endometrium or with RIF

**Fig. 7:**
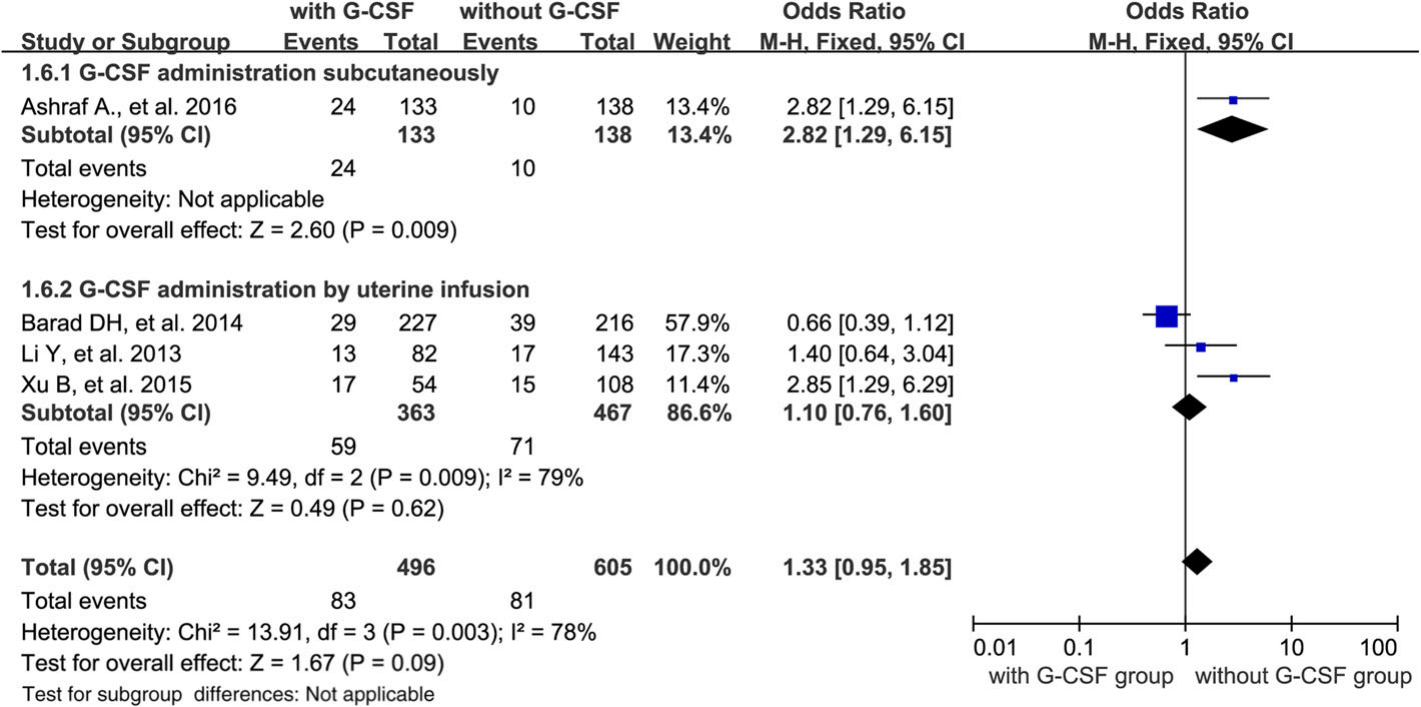
Forest plots showing the results of meta-analysis of studies evaluating the effect of G-CSF via different administration routes on embryo implantation rate after ART cycles

Besides, we also carried out a subgroup analysis according to the route of G-CSF administration (subcutaneous injection *n* = 2; uterine infusion *n* = 4). When evaluating the effect of G-CSF administration on PR, two studies administrated G-CSF via subcutaneous injection and four studies implied G-CSF by uterine infusion. The results showed an increased PR when G-CSF was administrated via subcutaneous injection (OR 3.12; 95 % CI 1.67–5.81, *P* = 0.003), and a similar PR when G-CSF was given via uterine infusion (OR 1.43; 95 % CI 0.91–2.24, *P* = 0.12) (Fig. 6). When evaluating the IR in cycles with different routes of G-CSF administration, one study used G-CSF subcutaneously and three studies applied G-CSF via uterine infusion. The result showed that there was no difference in IR when G-CSF was administrated via uterine infusion (OR 1.10; 95 % CI 0.76–1.60, *P* = 0.62) and the only one study used G-CSF subcutaneously showed an increased IR when G-CSF was administrated (Fig. 7).

All of the included studies have got good marks when the Newcastle-Ottawa Quality Assessment Scale was used (not shown). The funnel plots of meta-analysis, which evaluating the effect of G-CSF administration on PR/IR, was symmetrical and suggested that there was no publication bias (Additional file 1: Figure S1, Additional file 2: Figure S3, Additional file 3: Figure S4, Additional file 4: Figure S5 and Additional file 5: Figure S6). However, the studies showed modest publication bias when assess the effect of G-CSF administration on embryo IR (Additional file 6: Figure S2).

## Discussion

For all we know, the present study is the first systematic review and meta-analysis which assess the effect of G-CSF administration on the PR and IR after ART. Many studies have attempted to explore the role of G-CSF administration in IVF / ICSI / FET treatment cycles. Several studies found G-CSF administration have positive effect on the outcome after ART [15, 16, 20–22], while other studies did not show an improved outcome after ART with G-CSF administration [17, 18, 23]. In the present review and meta-analysis, 6 and 4 studies were included to evaluate the effect of G-CSF administration on the PR and IR, respectively.

The results have demonstrated that the G-CSF administration benefit the PR after ART cycles either with thin endometrium or RIF. The pooled OR was 2.03 for PP (95 % CI 1.19–3.46). IR showed an increased tendency with OR 1.59 (95 % CI 0.74–3.41), and there was no significantly difference between G-CSF group and non-G-CSF group.

In general, an adequate endometrial thickness above a threshold is necessary for embryo implantation. Thin endometrium always leads to embryo implantation failure. Besides, many couples still remain unsuccessful after several IVF attempts even if with normal endometrial thickness. With the deepening of understanding, researchers realized the important role of G-CSF in reproduction [25–31].

Gleicher and his co-authors reported the successful application of G-CSF in the treatment of thin endometrium for the first time in 2012. This paper reported four patients with thin endometrium conceived successfully after receiving G-CSF administration via uterine infusion. Subsequently many cohort studies were carried out to evaluate the effect of G-CSF administration on the outcome of ART cycles with thin endometrium or RIF, and came into controversial conclusions [15–18, 20–23].

It is well known that G-CSF, as a kind of cytokines, could stimulate the hematopoietic progenitor cells to proliferate and differentiate. And recently many researchers have found that G-CSF is likely associated with the reproductive system functions of females, for example follicular development, ovulation, ovarian response to stimulation, and establishment and maintenance of pregnancy [27, 32–36].

Most studies and our present meta-analysis demonstrated that G-CSF administration have beneficial effect on the clinical outcome after embryo transplantation. The possible explanations for the beneficial effect of G-CSF on the outcome of ART were as follows:

Firstly, G-CSF, which is a glycoprotein of growth factor family, has been found to regulate endometrial growth, and play a role in the genesis of early endometriotic lesions [37]. Another study showed that G-CSF would exert a direct effect on endometrial epithelial cell proliferation [38]. Additionally, G-CSF may stimulate the endometrial stem cells or mobilize bone marrow stem cells, and improve the development of endometrium [13, 20, 39]. To investigate the influence of G-CSF administration on the proliferation and differentiation of endometrial stromal cells, a study by Tanaka et al [32] found that G-CSF administration could induce the human endometrial stromal cells to be decidualization via cAMP-mediation in both autocrine and paracrine ways. So the G-CSF could expand the endometrial thickness and improve the endometrial receptivity.

Secondly, G-CSF and its receptor were expressed in both the endometrium and the fetomaternal interface [32, 40, 41]. Both fetal chorionic villous and maternal decidual tissues could secrete G-CSF during the first trimester. One study by Salmassi et al found infertile women who become pregnant have an increased level of serum G-CSF compared with women who without pregnancy, and concluded that G-CSF have a key role in the pregnancy achievement /maintenance [29]. And another study by Rahmati et al. demonstrated that infertile women with RIF have a significantly lower level of G-CSF receptors at the maternal-fetal interface, and G-CSF administration would be increase the expression of G-CSF receptors [26]. Local G-CSF administration significantly increased the expression of CD16, CD56, and LIF, which enhance the chance of pregnancy [18]. One study showed an improved implantation and pregnancy rates, and believed that local infusion of G-CSF was both chemical stimulation and mechanical stimulation, which lead to the secretion of endogenous cytokines and the activation of the endocrine paracrine pathways [18].

Thirdly, G-CSF might affect reproduction, implantation, and pregnancy through several possible mechanisms. G-CSF has been shown to induce the trophoblasts proliferation, invasion and maintenance during pregnancy [30, 31]. G-CSF also plays a key role during the embryo implantation process. G-CSF was involved in modulating genes, which were associated with adhesion of embryo, cell migration, tissue remodeling and angiogenesis. All of these processes are necessary for embryo implantation and placentation [26].

Lastly, successful pregnancy can be seen as an immune challenge to the maternal because the embryo was semi-allogenic. G-CSF might be induce appropriate modification which agree the immune tolerance in pregnancy; G-CSF switches the T cell cytokine secretion profile to the Th-2 responses, and promotes the differentiation of dendritic cell and regulatory T cell [42], which are important parts of the immunoregulatory events that occur before and after the implantation in the uterus [27].

Whereas some studies failed to find beneficial effect of G-CSF. We think the possible reasons were: first, the small sample size, and the less number of cycles. Second, the relatively low dose of G-CSF administrated. Third, once time of G-CSF administration. We supposed that the treatment effect would be obvious if the dose and the frequency of G-CSF administration were increased.

The ideal route of G-CSF administration has not been identified yet. Previous studies have used G-CSF via either subcutaneous injection or intrauterine infusion. In order to explore which route was better, we evaluated the effect on outcome with different routes of G-CSF administration. We found that G-CSF administrated subcutaneously resulted in significantly increased PR [OR 3.12 (95 % CI 1.67–5.81)] and IR [OR 2.82 (95 % CI 1.29–6.15)], whereas G-CSF administrated via uterine infusion had no beneficial effect on the PR [OR 1.42 (95 % CI 0.91–2.24)] and IR [OR 1.10 (95 % CI 0.76–1.60)] after ART. But the exact reason for this phenomenon is not clear.

As far as the strength, the meta-analysis resulted in a more accurate estimation with the pooled ORs value than single study. The pooled results of included studies indicated that G-CSF administration has a beneficial effect on pregnancy and implantation after IVF/ICSI/FET cycles with thin endometrium or RIF. When evaluating the effect of G-CSF administration on pregnancy, six studies were included, and the combined OR was above one with 95 % CI 1.19–3.46. While evaluating the effect of G-CSF administration on implantation, the combined OR showed an increased trend in IR but the difference had no significances (95 % CI 0.74–3.41).

Besides, there were also some limitations. A major limitation of the present study was the high level of heterogeneity among these included studies’ characteristics: different study object (cycle with thin endometrium / RIF), different treatment types (IVF / ICSI / FET) and different routes (subcutaneous injection / intrauterine infusion) and dose of G-CSF administration. Besides, small number of study subjects in the literature and lack of adjustment for meaningful confounders were all the flaws of the present study. Notwithstanding these drawbacks, the present systematic review and meta-analysis provides a valuable summary of the results of scientific publications so far.

## Conclusion

The present meta-analysis and systematic review suggested that G-CSF administration has beneficial effect on the clinical pregnancy outcome after IVF/ICSI/FET cycles. In spite of the small number of studies included and the variable characteristics of these studies, we suggest that administration of G-CSF subcutaneously would be an optimal treatment for those suffering thin endometrium or RIF. Further cohort studies are required to explore the mechanisms undergone the effect and investigate the best route and dose of G-CSF administration.

## Additional files

Additional file 1: Figure S1. Funnel plot of analysis for the effect of G-CSF administration on pregnancy rate, showing the results of Eggers to assess publication bias. (TIF 293 kb) Additional file 2: Figure S3. Funnel plot of analysis for the effect of G-CSF administration on pregnancy rate in thin endometrium or RIF cycles, showing the results of Eggers to assess publication bias. (TIF 346 kb) Additional file 3: Figure S4. Funnel plot of analysis for the effect of G-CSF via different administration routes on pregnancy rate, showing the results of Eggers to assess publication bias. (TIF 364 kb) Additional file 4: Figure S5. Funnel plot of analysis for the effect of G-CSF administration on embryo implantation rate in thin endometrium or RIF cycles, showing the results of Eggers to assess publication bias. (TIF 338 kb) Additional file 5: Figure S6. Funnel plot of analysis for the effect of G-CSF via different administration routes on embryo implantation rate, showing the results of Eggers to assess publication bias. (TIF 354 kb) Additional file 6: Figure S2. Funnel plot of analysis for the effect of G-CSF administration on embryo implantation rate, showing the results of Eggers to assess publication bias. (TIF 301 kb)

## Abbreviations

ART: Assisted reproductive technology
CI: Confidence interval
FET: Frozen embryo transfer
G-CSF: Granulocyte colony stimulating factor
ICSI: Intracytoplasmic sperm injection
IR: Implantation rate
IVF: In vitro fertilization
OR: Odds ratio
PR: Pregnancy rate
RIF: Repeated IVF failure
