# Supplementary figures and images for: Whether G-CSF administration has beneficial effect on the outcome after assisted reproductive technology? A systematic review and meta-analysis
Source: Reprod Biol Endocrinol. 2016 Sep 22;14:62. doi: 10.1186/s12958-016-0197-2 (PMC5034435; doi:10.1186/s12958-016-0197-2)

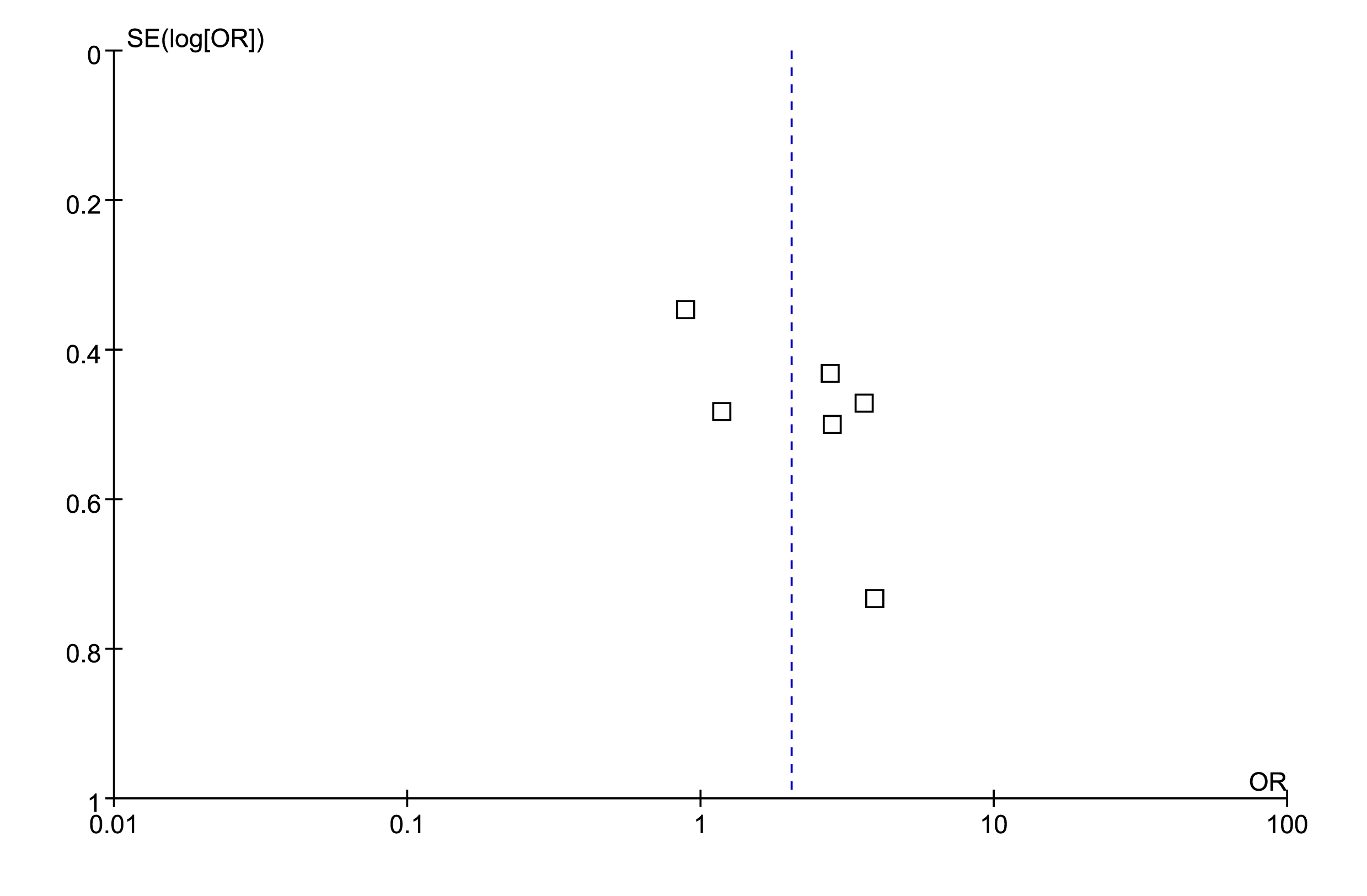

Supplement: Additional file 1: Figure S1. — Funnel plot of analysis for the effect of G-CSF administration on pregnancy rate, showing the results of Eggers to assess publication bias. (TIF 293 kb) [file 12958_2016_197_MOESM1_ESM.tif]

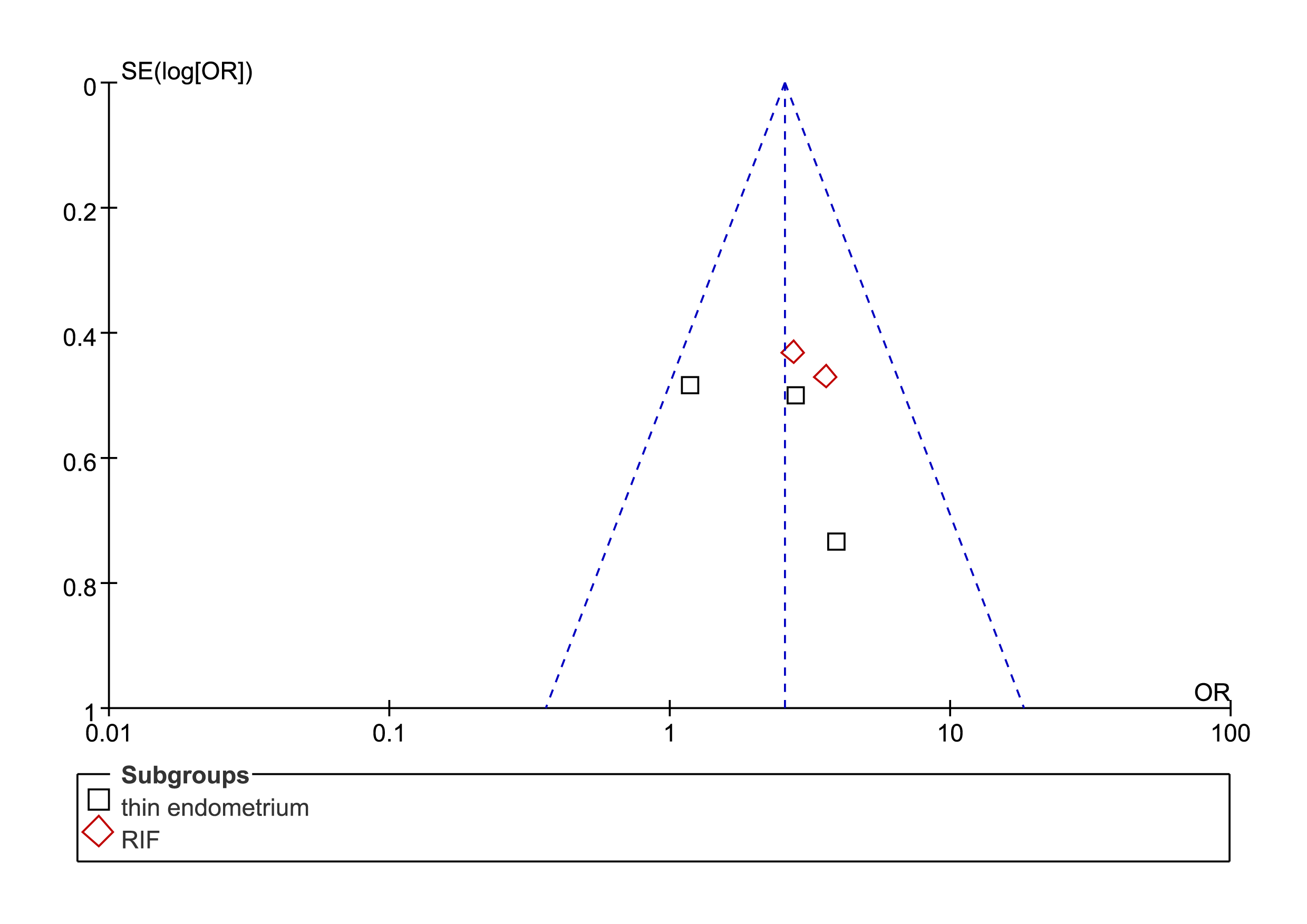

Supplement: Additional file 2: Figure S3. — Funnel plot of analysis for the effect of G-CSF administration on pregnancy rate in thin endometrium or RIF cycles, showing the results of Eggers to assess publication bias. (TIF 346 kb) [file 12958_2016_197_MOESM2_ESM.tif]

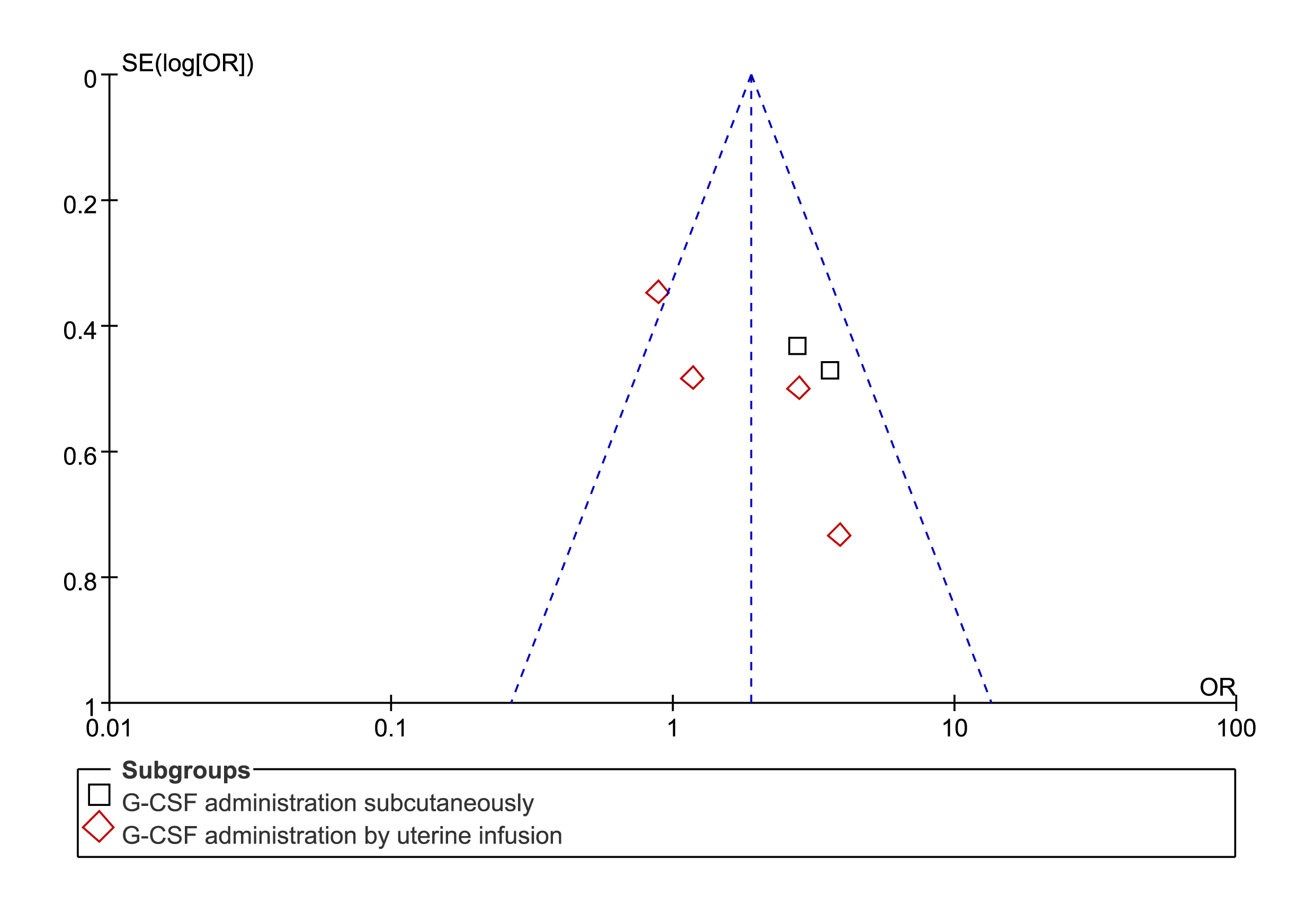

Supplement: Additional file 3: Figure S4. — Funnel plot of analysis for the effect of G-CSF via different administration routes on pregnancy rate, showing the results of Eggers to assess publication bias. (TIF 364 kb) [file 12958_2016_197_MOESM3_ESM.tif]

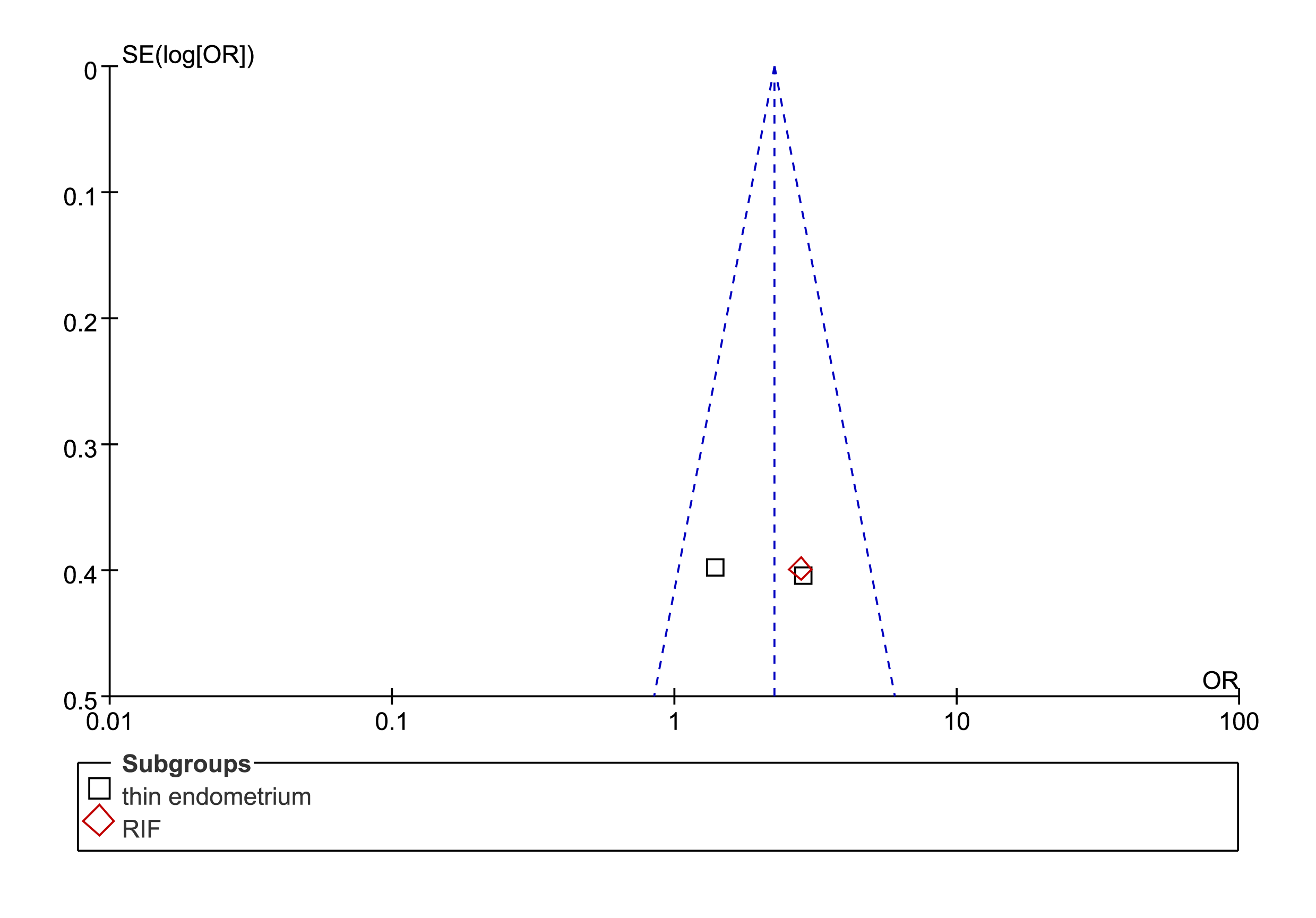

Supplement: Additional file 4: Figure S5. — Funnel plot of analysis for the effect of G-CSF administration on embryo implantation rate in thin endometrium or RIF cycles, showing the results of Eggers to assess publication bias. (TIF 338 kb) [file 12958_2016_197_MOESM4_ESM.tif]

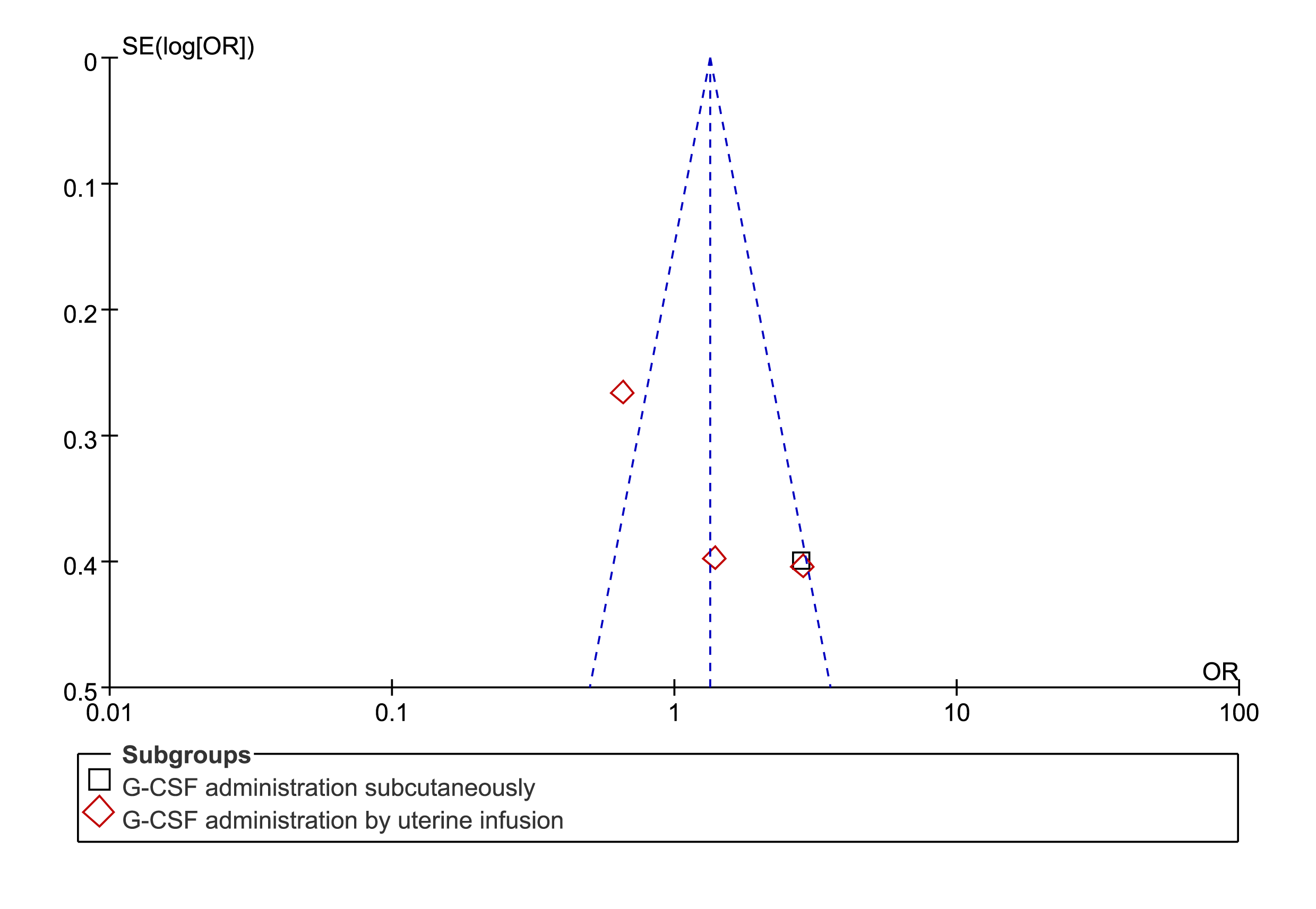

Supplement: Additional file 5: Figure S6. — Funnel plot of analysis for the effect of G-CSF via different administration routes on embryo implantation rate, showing the results of Eggers to assess publication bias. (TIF 354 kb) [file 12958_2016_197_MOESM5_ESM.tif]

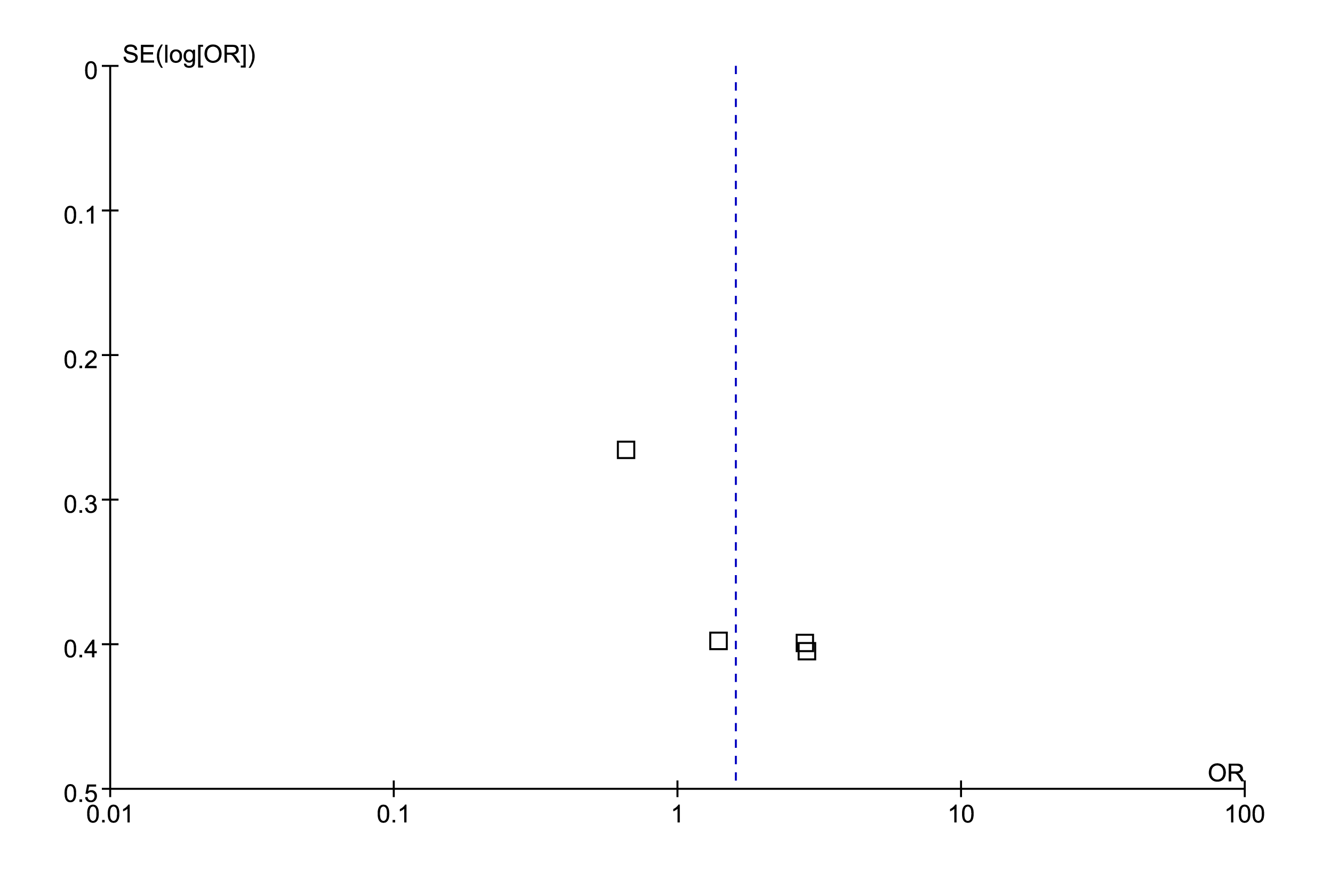

Supplement: Additional file 6: Figure S2. — Funnel plot of analysis for the effect of G-CSF administration on embryo implantation rate, showing the results of Eggers to assess publication bias. (TIF 301 kb) [file 12958_2016_197_MOESM6_ESM.tif]
